# The Microbial Community Associated with *Rhizostoma pulmo*: Ecological Significance and Potential Consequences for Marine Organisms and Human Health

**DOI:** 10.3390/md18090437

**Published:** 2020-08-21

**Authors:** Loredana Stabili, Lucia Rizzo, Lorena Basso, Marinella Marzano, Bruno Fosso, Graziano Pesole, Stefano Piraino

**Affiliations:** 1 Department of Biological and Environmental Sciences and Technologies, University of Salento, Via Prov.le Lecce Monteroni, 73100 Lecce, Italy; lorena.basso@unisalento.it (L.B.); stefano.piraino@unisalento.it (S.P.); 2 Institute of Water Research of the National Research Council, S.S. di Taranto, Via Roma 3, 74123 Taranto, Italy; 3 Integrative Marine Ecology, Stazione Zoologica Anton Dohrn, Villa Comunale, 80121 Napoli, Italy; 4 Istituto di Biomembrane, Bioenergetica e Biotecnologie Molecolari (IBIOM), CNR, 70126 Bari, Italy; b.fosso@ibiom.cnr.it (B.F.); g.pesole@ibiom.cnr.it (G.P.); 5 Dipartimento di Bioscienze, Biotecnologie e Biofarmaceutica, Università degli Studi di Bari “Aldo Moro”, 70121 Bari, Italy; 6 CoNISMa, Piazzale Flaminio 9, 00196 Rome, Italy

**Keywords:** scyphomedusae, 16S amplicon sequencing analysis, high-throughput sequencing, taxonomic microbial diversity, BIOLOG system

## Abstract

Jellyfish blooms are frequent and widespread in coastal areas worldwide, often associated with significant ecological and socio-economic consequences. Recent studies have also suggested cnidarian jellyfish may act as vectors of bacterial pathogens. The scyphomedusa *Rhizostoma pulmo* is an outbreak-forming jellyfish widely occurring across the Mediterranean basin. Using combination of culture-based approaches and a high-throughput amplicon sequencing (HTS), and based on available knowledge on a warm-affinity jellyfish-associated microbiome, we compared the microbial community associated with *R. pulmo* adult jellyfish in the Gulf of Taranto (Ionian Sea) between summer (July 2016) and winter (February 2017) sampling periods. The jellyfish-associated microbiota was investigated in three distinct compartments, namely umbrella, oral arms, and the mucus secretion. Actinobacteria, Bacteroidetes, Chlamydiae, Cyanobacteria, Deinococcus-Thermus, Firmicutes, Fusobacteria, Planctomycetes, Proteobacteria, Rhodothermaeota, Spirochaetes, Tenericutes, and Thaumarchaeota were the phyla isolated from all the three *R. pulmo* compartments in the sampling times. In particular, the main genera *Mycoplasma* and *Spiroplasma*, belonging to the class Mollicutes (phylum Tenericutes), have been identified in all the three jellyfish compartments. The taxonomic microbial data were coupled with metabolic profiles resulting from the utilization of 31 different carbon sources by the BIOLOG Eco-Plate system. Microorganisms associated with mucus are characterized by great diversity. The counts of culturable heterotrophic bacteria and potential metabolic activities are also remarkable. Results are discussed in terms of *R. pulmo* ecology, the potential health hazard for marine and human life as well as the potential biotechnological applications related to the associated microbiome.

## 1. Introduction

Jellyfish represent ubiquitous components of world oceans. Many species are characterized by sudden rapid outbreaks (blooms) in alternation with rarity periods, a natural feature of healthy pelagic ecosystems [1,2]. However, natural fluctuations of jellyfish abundance may be changed in coastal waters by the interactive impacts of multiple anthropogenic stressors including habitat modification, overfishing, species translocation, eutrophication, and/or climate change with consequent ocean warming [1,3,4,5,6,7]. Regardless of the uncertainty and contrasting evidence of natural vs anthropogenic global trends and bloom drivers [8,9,10], jellyfish populations may bloom in a relatively short period of time attaining huge biomasses in some coastal areas as well as in large marine ecosystems [11,12]. Increased attention has been addressed to jellyfish outbreaks in the early 1980s when massive outbreaks of *Pelagia noctiluca* in the Mediterranean were responsible of injuries to tourists and fishermen. In the last 50 years, jellyfish are apparently on the rise in several coastal areas, including the Mediterranean Sea, where jellyfish blooms periodically become an issue to marine and maritime human activities. Some studies have highlighted the negative impact of jellyfish blooms on human welfare in relation to the presence of venoms in specialized cnidarian cells armed with stinging organelles, called cnidocysts, introducing venomous proteinaceous and non-proteinaceous substances with cytolytic, cytotoxic, and enzymatic properties [13,14]. In this framework, recurrent massive jellyfish outbreaks constitute a significant economic issue to the Mediterranean countries [15]. Jellyfish outbreaks may also have broad ecological consequences related to their top-down control on zooplankton communities or as a resource for vertebrate predators, so affecting pelagic food webs at different trophic levels [16,17,18,19]. Jellyfish are also known to produce bottom-up influences on primary production as well as on microbial and phytoplanktonic assemblages [20,21,22,23]. Further, gelatinous particulate organic matter derived from decaying jellyfish (jelly-falls) as a post-bloom process is known as a powerful process of exporting large surface carbon production downwards to the benthic systems [24]. Jellyfish blooms also exert social impacts on other human activities including fisheries and aquaculture [5,10,25,26,27,28,29]. Finfish mariculture may be particularly endangered by blooms of jellyfish stingers, which can enter fish cages producing skin lesions, gill epithelial damage and metabolic distress on reared fish, leading to mass mortality [15,30,31]. Jellyfish may also function as carriers of microbial pathogens, as for the bacterium *Tenacibaculum maritimum* isolated from the jellyfish *Pelagia noctiluca*, responsible of severe gill diseases of farmed fish [29,32,33].

Cnidarians have many microorganisms associated (epibiotic or symbiotic) with their tissues [34]. As reported by Tinta et al. [35] early reports on microorganisms associated with jellyfish resulted as corollary observations, whereas primary targets of research were jellyfish [33,36,37]. Later studies, focusing on the relationships between microorganisms and their host organisms, addressed more specific issues on the composition and ecological role of jellyfish-associated microbial communities [35,38,39]. In the last decades, many studies revealed the role played by microorganisms in coral life histories, particularly the dynamic assemblage formed by the coral host, its endosymbiotic dinoflagellates, and a number of accompanying microorganisms, i.e., the coral holobiont [40,41,42]. Further studies focused on bacteria associated to outer surfaces of cnidarian epithelia belonging to different taxa and life stages demonstrating their involvement in several crucial potential roles, such as nitrogen fixation [43], antibiotics synthesis [44,45] organic compounds decomposition [46], primary defense against pathogens [47], or modulation of contractile activities [48].

So far, a limited number of studies explored different types of interactions between marine microbial communities and scyphozoan jellyfish, from host-microbiome interactions to quali-quantitative changes of microbial composition sampled across different life stages, medusa body parts, water samples, mostly on common semeostome jellyfish [22,23,35,38,49,50,51,52,53,54,55]. To our knowledge, *Mastigias papua*, *Cotylorhiza tuberculata,* and *Rhizostoma pulmo* are the only rhizostome jellyfish species previously investigated with respect to their associated microbial community [39,50,51,55,56].

*Rhizostoma pulmo* is an endemic Mediterranean jellyfish with a whitish dome-shaped umbrella that can reach up to 50–60 cm in diameter, eight fleshy oral arms, and a tentacle-less, blue-colored umbrella edge [57]. It displays high tolerance to changes in salinity and temperature conditions and is common in eutrophic areas [58]. It is widely distributed in the Mediterranean basin, from Spain to Marmara Sea, and in the Black Sea [59,60]. In the last years, the number of bloom sightings (>10 ind/m^2^) of *R. pulmo* has been increased along the Mediterranean coasts [1]. In the Ionian Sea, along the Taranto Gulf coasts, *R. pulmo* has been present regularly since 2005, getting high abundances from July to October [39]. Recently, using an ultra-light aerial survey, a remarkable *R. pulmo* outbreak characterized by over 48,000 ind/km^2^ and a biomass assessment of ~300 t/km^2^ was reported along the southwestern shores of the Gulf of Taranto [61].

Many jellyfish species, including *R. pulmo,* represent a potential exploitable source of bioactive compounds in nutritional, nutraceutical, cosmeceutical, and pharmacological applications on account of their high biomasses and their associated microbiome [62,63,64,65,66]. Taking advantage from ongoing research on the microbial assemblages associated to common Mediterranean jellyfish [22,39], the present study aimed to investigate the microbiome associated with different fractions (umbrella, oral arms, and secreted mucus) of jellyfish at the lowest sea surface temperature values (typically February in the Mediterranean [67]), and to compare it with that already described during the warmest months (July–August) from the same jellyfish species, *R. pulmo,* and locality in the Northern Ionian Sea (Gulf of Taranto, SE Italy) [39]. By the integration of culture-dependent methods with a high-throughput amplicon sequencing (HTS) approach, we gained insight into *R. pulmo* associated bacteria and the body compartment-specific bacterial colonization. The role of *Rhizostoma pulmo* as vector for spreading the jellyfish-associated microbiome was investigated, with relevance to the potential consequences for marine organisms and human health and the ecological significance of jellyfish-associated microorganisms.

## 2. Results

### 2.1. Bacterial Enumeration: Comparative Analysis

**Figure 1:**
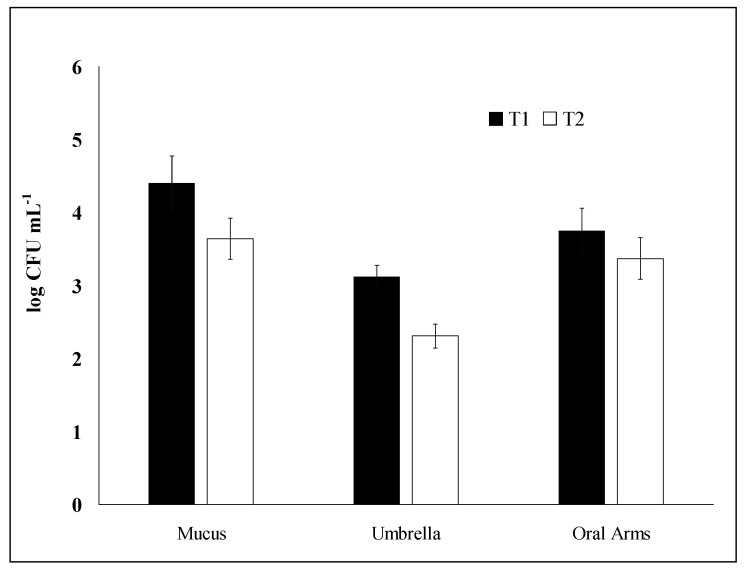
Culturable heterotrophic bacterial abundance (as colony forming unit CFU counts) associated with *Rhizostoma pulmo* compartments: Mucus (M) umbrella (U), and oral arms (A) in July (T1) and February (T2). Data are reported as mean values ± S.E.

The highest concentration of culturable heterotrophic bacteria associated with the three *R. pulmo* compartments has been found in the mucus (M) in T1 (July 2016) corresponding to a mean value of 2.5 × 10^4^ colony forming unit (CFU)/mL while the lowest concentration has been recorded in the umbrella (U) in T2 (February 2017) with a mean value of 2 × 10^2^ CFU/mL (Figure 1).

PERMANOVA (Permutational Multivariate Analysis of Variance) analysis showed the significant C × T interaction term (Table 1) and post-hoc pairwise tests (Table 2) underlined significant differences of culturable heterotrophic bacterial abundances among compartments in T1 and partly in T2, when bacterial abundances associated with mucus and arms did not show significant differences.

### 2.2. Microbial Profiles Related to Potential Carbon Sources Utilization: Comparative Analysis

By the BIOLOG ECO plate system significant differences in the potential utilization of the 31 carbon sources by microbial population associated with the different *R. pulmo* compartments in T1 and T2 were evidenced, as revealed by the significant C × T interaction term (Table 1) and the subsequent pairwise analyses (Table 2). The highest metabolic activity, measured in terms of growth over a range of carbon substrates, was recorded for the heterotrophic microorganisms associated with mucus (M) and the lowest activity for the microorganisms associated with umbrella (U) both in T1 and T2 (Table 3).

In both sampling months, mucus associated bacteria degraded 10 common substrates and associated bacteria of arms degraded 4 common substrates. In particular the d-galacturonic carboxylic acid was degraded by both the microorganisms associated with mucus and arms. The heterotrophic microorganisms associated with umbrella were able to degrade 3 substrates in T1 and only one substrate in T2 (Table 3).

**Figure 2:**
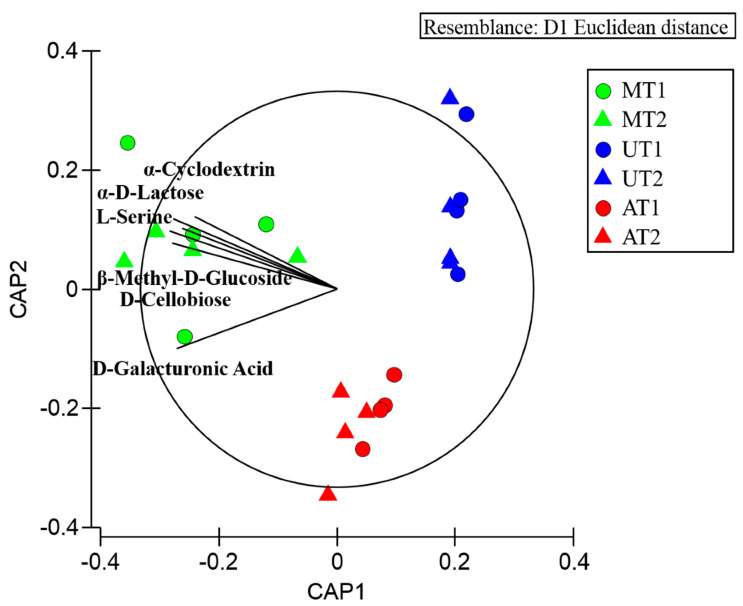
Canonical analysis of principal coordinates (CAP) plot showing the metabolic utilization of 31 different carbon sources by the microbial community associated with mucus (M), umbrella (U), and oral arms (A) and of *R. pulmo* in July (T1) and February (T2). Vectors are proportional to the Pearson correlation of the carbon source variables.

The CAP (canonical analysis of principal) plot showed a segregation across jellyfish compartments in T1 and T2 (Figure 2), which is mainly due to d-Glucosaminic Acid degraded by microorganisms associated with mucus and arms, and to α-Cyclodextrin, α-d-Lactose, l-Serine, β-Methyl-d-Glucoside, d-Cellobiose, degraded by the mucus associated microorganisms.

### 2.3. Microbial Diversity: Comparative Analysis

Twenty-four libraries (four pools from each jellyfish compartment per each sampling period, T1 and T2) of dual indexed amplicons of 420 bp related to the V5-V6 hyper-variable of the 16S rRNA gene were successfully sequenced in two MiSeq platform run, using a 2 × 250 bp paired-end (PE) sequencing strategy. All sequenced samples generated reads of high quality with the expected length of 250 bp. About 80% and 92% (Standard Deviation, SD 3.38 and 3) of the produced PE reads, in the first and second sequencing run respectively, were retained as ASVs (Amplicon Sequence Variants), following the denoising procedure. In particular, 456 (T1 = 311, T2 = 247), 306 (T1 = 183, T2 = 205), and 271 (T1 = 146, T2 = 185) were inferred for samples of arms, mucus and umbrella, respectively.

**Figure 3:**
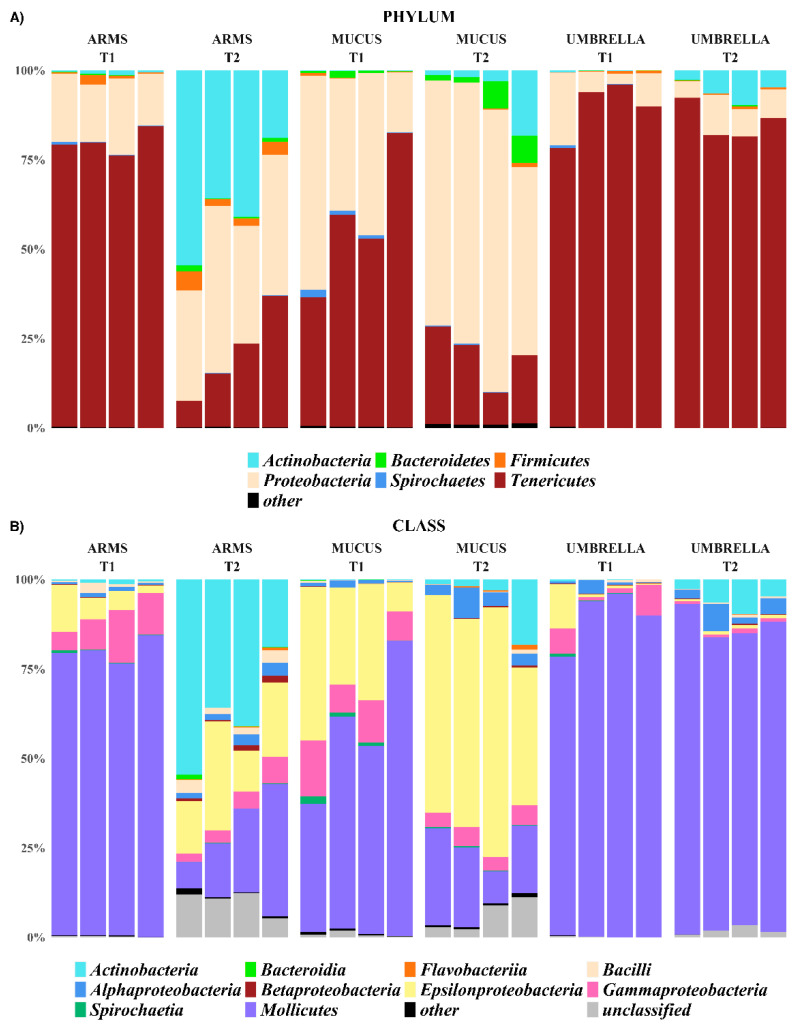
Stacked bar-plot of the relative abundances at phylum (**A**), and class (**B**) level of all the arms, mucus, and umbrella samples at the T1 (July 2016) and T2 (February 2017) sampling times. In particular, only the taxa with a relative abundance equal or higher than 1% were plotted, otherwise were collapsed into “Other”.

**Figure 4:**
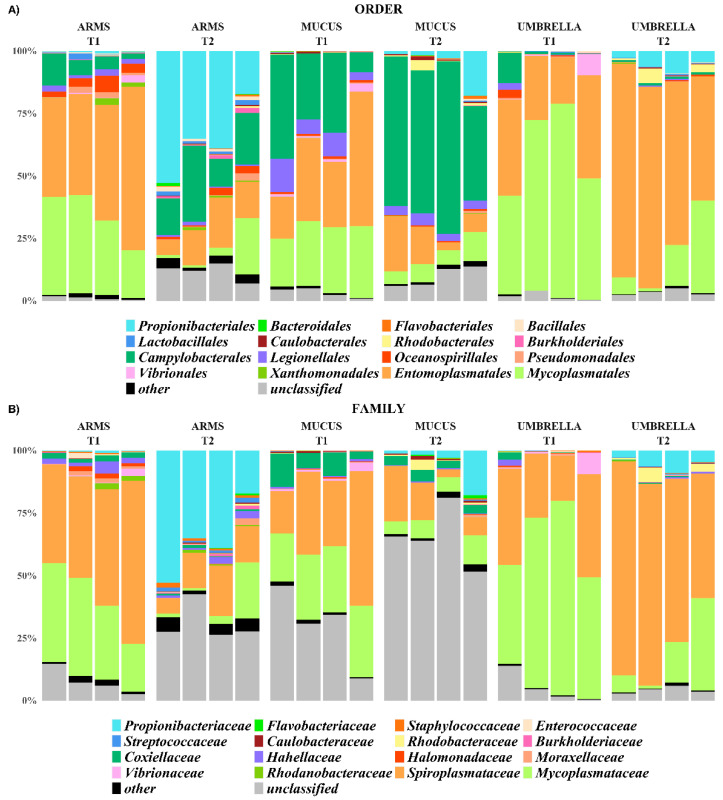
Stacked bar-plot of the relative abundances at order (**A**) and family (**B**) level of all the arms, mucus and umbrella samples at the T1 (July 2016) and T2 (February 2017) sampling times. In particular, only the taxa with a relative abundance equal or higher than 1% were plotted, otherwise were collapsed into “Other”.

**Figure 5:**
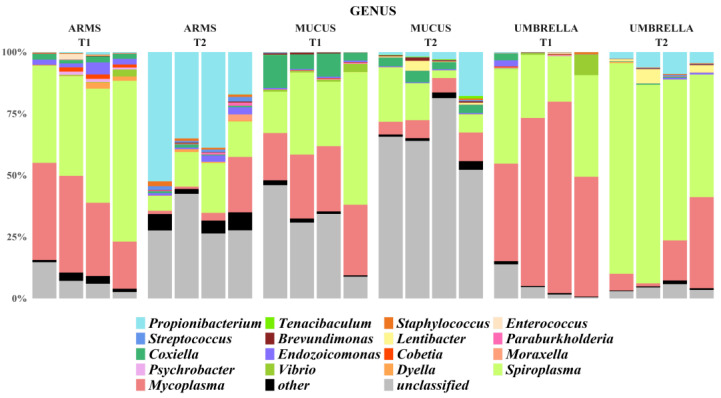
Stacked bar-plot of the relative abundances at genus level of all the arms, mucus, and umbrella samples at the T1 (July 2016) and T2 (February 2017) sampling times. In particular, only the taxa with a relative abundance equal or higher than 1% were plotted, otherwise were collapsed into “Other”.

The relative abundances of identified taxa, from phylum to genus level, were reported as stacked bar plot for each analyzed sample (Figure 3, Figure 4 and Figure 5). In particular, only taxa with a relative abundance (RA) equal or higher than 1% were plotted, otherwise were collapsed into “Other”. Actinobacteria, Bacteroidetes, Chlamydiae, Cyanobacteria, Deinococcus-Thermus, Firmicutes, Fusobacteria, Planctomycetes, Proteobacteria, Rhodothermaeota, Spirochaetes, Tenericutes, and Thaumarchaeota were the phyla isolated from all the three *R. pulmo* compartments in the sampling times.

The phyla Tenericutes (57.6 ± 19.3% in mucus, 79.8 ± 0.0% in arms and 89.4 ± 8.0% in umbrella compartments, in T1; 19.4 ± 7.8% in mucus, 20.6 ± 12.6% in arms and 85.6 ± 5.0% in umbrella compartments, in T2) and Proteobacteria (39.8 ± 18.0% in mucus, 17.7 ± 3.1% in arms and 9.6 ± 7.6% in umbrella, in T1; 68.3 ± 11.3% in mucus, 37.4 ± 7.1% in arms and 7.9 ± 2.7% in umbrella compartments, in T2) were common in all the three compartments and the sampling periods (RA > 1%). Within the phylum Tenericutes, which was more represented (*p <* 0.05) in T1-related jellyfish samples than in T2-related samples and in particular associated to umbrella (*p <* 0.001), the Mollicutes, with the genera *Mycoplasma* and *Spiroplasma* (o. Mycoplasmatales, f. Mycoplasmataceae; o. Entomoplasmatales, f. Spiroplasmataceae, respectively), was the only assigned class. At species level, *Mycoplasma faucium* was identified (RA > 1%) in all the analyzed samples. The phylum Proteobacteria was dominated by the following three classes: Alphaproteobacteria, Epsilonproteobacteria and Gammaproteobacteria (Figure 3). The Alphaproteobacteria appeared associated, with RA > 1%, to mucus and umbrella in T1, while in T2 it was the only class present in all three compartments with the following calculated values 4.66 ± 2.64% in mucus, 2.44 ± 1.04% in arms and 4.11 ± 2.60% in umbrella compartments, respectively. Instead, the Epsilonproteobacteria and Gammaproteobacteria were the most abundant (27.6 ± 14.6% and 10.8 ± 3.6% in mucus, 6.7 ± 4.6% and 10 ± 4.1% in arms, 3.6 ± 5.9% and 4.5 ± 4.0% in umbrella, respectively) in the three compartments collected over the T1 period and assigned to mucus and arms, with RA > 1%, in T2. The Caulobacteraceae and Rhodobacteraceae for Alphaproteobacteria and the Coxiellaceae, Hahellaceae, Halomonadaceae, Moraxellaceae, Rhodanobacteraceae, and Vibrionaceae for Gammaproteobacteria were taxonomically identified at the family level, with a different distribution among the three compartments and also between the T1 and T2 sampling periods. This, except for the Coxiellaceae (T1: 8.0 ± 4.5%; T2: 3.6 ± 2.0%), represented by the genus *Coxiella* (T1: 3.6 ± 0.0%; T2: 1.6 ± 0.0%)*,* and Hahellaceae (T1: 1.33 ± 0.0%, T2: 0.82 ± 0.0%), with the genus *Endozoicomonas* (T1: 1.31 ± 0.0%; T2: 0.82 ± 0.0%)*,* were the most abundant taxa associated with mucus (*p <* 0.05) and samples of arms, respectively, in both the sampling periods. In particular, *Endozoicomonas atrinae* was identified in the arms. On the contrary, the umbrella and the mucus compartments were characterized by *Vibrio anguillarum* (f. Vibrionaceae) at T1 and by *Lentibacter algarum* (f. Rhodobacteraceae) at T2. at T1, in addition to Hahellaceae, the families Halomonadaceae, Moraxellaceae, and Rhodanobacteraceae were assigned to the arms (A).

The phyla Actinobacteria, Firmicutes, Spirochaetes and Bacteroidetes were associated, with RA > 1%, only to specific compartments and/or sampling period. For example, the Actinobacteria were less represented (*p <* 0.001) in all the T1-related jellyfish samples than in T2-related samples (T2: 6.1 ± 8.1% in mucus, 37.5 ± 14.8% in arms and 5.9 ± 3.0% in umbrella; T1: 0.14 ± 0.04% in mucus, 0.73 ± 0.45% in arms and 0.18 ± 0.19% in umbrella). The same trend has been observed at a deeper taxonomic level, where the family Propionibacteriaceae, represented by the genus *Propionibacterium*, was more abundant in T2 (February) than T1 (July), especially in the arms. The phyla Firmicutes was associated with arms, both at T1 and T2, Spirochaetes to mucus samples at T1 and Bacteroidetes to mucus samples (*p* < 0.001) at T2. Within Firmicutes, the genera *Streptococcus* and *Staphylococcus* (order Lactobacillales and Bacillales, respectively) were found, with a RA > 1%, only in arms of T2. The other phyla were represented with relative abundance lower than 1% in all the analyzed samples.

Based on the ASVs counts generated by DADA2 (Divisive Amplicon Denoising Algorithm 2), the biodiversity of the jellyfish-associated microbial communities (in terms of richness and abundance) was estimated on the 24 pooled samples by two quantitative alpha diversity indices, the Shannon index (H’) and the Faith Phylogenetic index (PD). Comparable values of both H’ and PD between T1 and T2 were detected. Similarly, no significant differences were detected among all the three jellyfish compartments (mucus, arms and umbrella). Differently, within each of the T1 and T2 samplings, the H’ index values calculated from mucus and arms were similar to each other but both were statistically different to the umbrella compartment. A different trend in PD values measured in T1 and T2 was observed. In particular, while arms and mucus were statistically different in T2, they were not in T1.

**Figure 6:**
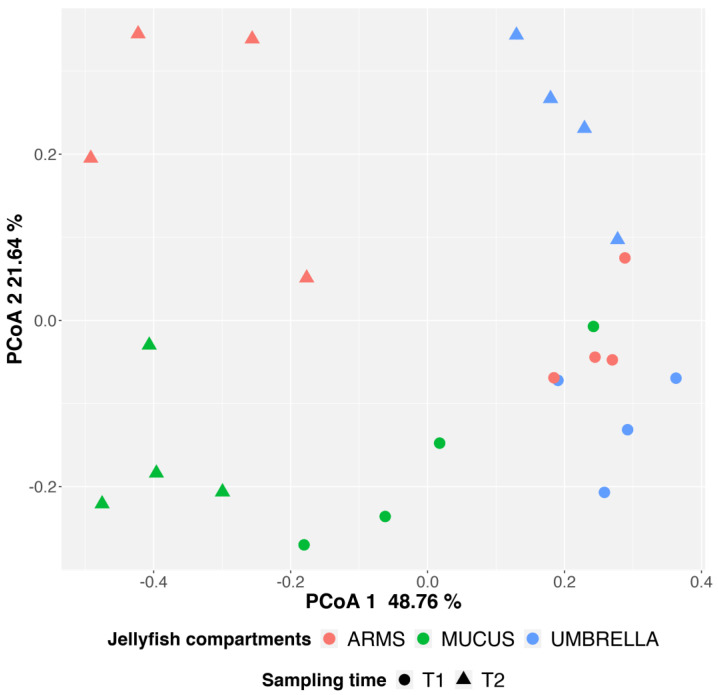
PCoA (Principal Coordinates Analysis) plots based on the based on Bray–Curtis Dissimilarity matrix. Points color and shape corresponds to jellyfish compartment (red ARMS, green MUCUS, and blue UMBRELLA) and sampling times (circles T1 and triangles T2).

The Bray–Curtis dissimilarity metric was applied to evaluate the beta diversity (i.e., the diversity between samples or proportional species turnover) and plotted as PCoA (Principal Coordinates Analysis) (Figure 6). In the PCoA plot, both the mucus and arms were clustered according to sampling time along the first component (48.76% of the observed data variability), but with a higher intra-group variability for T1 arms. Conversely, umbrella samples were localized on the left side of the plot and distributed according to sampling period along the second component (21.64%).

By using the PERMANOVA analysis, a significant statistical difference was observed between compartments and sampling periods (both *p* ≤ 0.001). In particular, the jellyfish compartments and the sampling period explained about 32% and 26% of the observed data variability. The SIMPER (Similarity Percentage) analysis allowed to identify the ASVs contributing to the dissimilarities between compartments and sampling time. Only ASVs obtaining a Permutation *p*-value ≤ 0.05 were considered. In particular, 87, 46 and 48 ASVs drove the dissimilarities between arms and mucus, mucus and umbrella, and arms and umbrella, respectively. 98 and 93 ASVs drove the Bray–Curtis dissimilarities in mucus and arms, while only 14 were identified for umbrella samples.

## 3. Discussion

In the present study, by a combination of 16S amplicon sequencing and culture-enrichment approaches, temporal differences between the microbial communities associated with *Rhizostoma pulmo* jellyfish during the warmest and coldest months of a solar year (July 2016–February 2017) were evaluated in term of taxonomic composition, abundance of culturable bacteria (expressed as CFU counts), and profiles of carbon sources utilization. While this study does not allow to understand the year-round microbiome composition, it gives a snapshot picture of two potentially opposite, temperature-dependent, jellyfish-associated microbial assemblages, so contributing to evaluate the role of jellyfish as “carrier” of microbial pathogens for humans or other marine organisms and/or as driver of the temporal microbial diversity. Changes of microbiome associated with different jellyfish species and geographical areas were previously investigated during episodic jellyfish blooms [22,23,38,39,68]. The present work aimed to ascertain whether a “core” of bacteria can be found to be associated with the “sea lung” or “barrel” jellyfish, *R. pulmo*, in both the warmest and coldest periods of the year. This species has been recently proposed as a novel, “blue growth” resource for nutritional, nutraceutical, and other biotechnological applications [61,62,64,69]. Previous researches dealt with the impact of jellyfish mucus release and biomass decay on bacterioplankton growth and community composition [70,71,72]. More recently, thanks to increasing use of next-generation technologies, a deeper understanding of the ecological consequences raised by invasive jellyfish blooms and their associated bacterial communities have been achieved [22,39,65,73].

The integrative approaches used here made possible to better understand the jellyfish–microbiome association by achieving a deep coverage of the prokaryotic communities. Umbrella (U) samples were characterized by a lower microbial diversity as demonstrated by both alpha and beta diversity analysis. Moreover, as shown by the SIMPER analysis, the temporal microbial composition in the jellyfish umbrella seems to be more similar, compared to those found in jellyfish arms (A) and mucus (M) in the two sampling times. Arms and mucus, indeed, were characterized by the most diverse microbial communities in terms of total observed ASVs, H’, and PD indexes. For both compartments, a larger variation of prokaryotes dwellers was observed in the two sampling times, as detected by the SIMPER analysis and supported also by the PCoA plot of the Beta diversity (Figure 6). In particular, arms were the jellyfish compartment the most sensitive to temporal changes. Regarding mucus it is well known that the large amount of mucus is secreted by the jellyfish in the water column; thus, mucus, on account of the high bacterial diversity and density recorded in the present study could be considered as a potential vector for environmental microorganisms including pathogens for humans and marine organisms. Cnidarians mucus is produced by secretory cells of epidermis and endoderm with important functions in the biology and survival of organisms, including protective and preventive role against infections [74,75]. The transferring of the collected food into the gastric cavities and the cleaning of small ciliated grooves of branched oral arms of rhizostomid jellyfish is related to mucus secretion. The release of mucus embedding clusters of stinging cells is also used as defense mechanism for several cnidarians, including *Cassiopea* spp. and *Rhizostoma pulmo* jellyfish ([65] Piraino, unpublished observation in [76]). Proteins, lipids, and a lower percentage of carbohydrates are the main components of jellyfish mucus matrix [77] making it a suitable substrate for several bacteria [35,39,52,65,73,78,79,80,81]. The microbial communities associated with the semeostome jellyfish *Aurelia aurita*, the scyphozoan *Mastigias* cf. *papua etpisoni* and the box jellyfish *Tripedalia* cf. *cystophora* were analyzed in different studies, identifying jellyfish as a host of bacterial associates [52,54,55]. In particular, the microbial community of jellyfish *Aurelia* sp. seems to be strictly host-specific and different from the bacterioplankton suspended in the surrounding water column [35,49,52,55]. In *Aurelia* sp., the microbiome associated with mucus is more variable compared to bacteria living in the gastric cavity, likely thanks to trapping properties of mannose and mucine glycan components of mucus [78,82].

In the present work, culturable methods, including the BIOLOG system, have been utilized to detect differences among microbiomes associated with different body compartments in the two sampling months (July 2016 vs February 2017). Although it is well known that the culture-based studies represent a limited compartment of total bacterial community [83,84,85], these techniques are also recognized as a crucial step for better integration of the physiological and ecological information [38,85,86]. The BIOLOG system has provided some pieces of information on the potential metabolic utilization of the 31 carbon sources by the microbial communities associated with the different examined compartments, showing that the umbrella microbiome utilized only few of the EcoPlate carbon sources in both the sampling months (July and February). This finding together with the lower culturable heterotrophic bacterial counts as well as with the lower microbial diversity of this compartment in comparison with the other ones leading to hypothesize a strict selectivity of specific microbial taxa for this body compartment. The microbial community associated with the oral arms showed an increase of the metabolic activities in February when compared with the metabolic potential observed in July, however, the increased bacterial counts and diversity is not recorded in this month. Further analysis will clarify the pattern of metabolic carbon sources utilization of microbiomes associated with this compartment. Finally, the microbial community associated with *R. pulmo* mucus exhibited the largest metabolic potential of carbon sources utilization in the two sampling months, as supported by the CAP plot showing the utilization of α-Cyclodextrin, α-d-Lactose, l-Serine, β-Methyl-d-Glucoside, d-Cellobiose, degraded by the mucus associated microorganisms. In particular, these substrata are utilized consistently over the time, suggesting that the jellyfish can produce some carbon sources that, in turn, select the mucus associated microbial communities based on metabolic traits, as already observed in literature [39,68]. The wide potential metabolic utilization of carbon sources by mucus associated bacteria of *R. pulmo* together with the observed high culturable bacterial counts and diversity supports the hypothesis that mucus may represent a suitable food source and “home site” for marine bacteria and viruses, as suggested by several researches [87,88,89,90].

A combination of the BIOLOG-EcoPlate selective test and 16S amplicon sequencing analyses shed light on the taxonomic diversity of the bacterial communities over the sampling times and jellyfish compartments. In particular, the main genera *Mycoplasma* and *Spiroplasma,* belonging to the class Mollicutes (phylum Tenericutes), have been identified in all the three jellyfish compartments in both sampling times. Mollicutes bacteria are known to occur as parasites on many eukaryotes [91], including algae [92,93] and many invertebrate taxa, such as bivalves [94], bryozoans [95], crustaceans [96], ctenophores [97], gastropods [93,98], and other cnidarians [50,51,52,99]. Some Mollicutes species are considered to be pathogens of metazoans [96,100,101], including humans [102]. These species may have a parasitic lifestyle, since they are characterized by small size and simple cell structure, lack of a cell wall, small genome, and simplified metabolic pathways [35]. Differently, the association between the cold-water coral *Lophelia pertusa* and *Mycoplasma corallicola* has been hypothesized as case of commensalism by Neulinger [103].

The first presence of *Spiroplasma* in jellyfish has been recognized in the semeostome *Pelagia noctiluca* by molecular methods [104], whereas a novel *Mycoplasma* strain was found associated both to the polyp and medusa stages of the moon jellyfish *Aurelia aurita* [52,55]. Viver et al. [50] found *Mycoplasma* spp. together with a supposed commensal *Spiroplasma*-like bacteria, with a genome smaller than known genomes of *Spiroplasma* spp., associated with the gastric cavity of the rhizostomid scyphomedusa *Cotylorhiza tuberculata*. In the present study, *Mycoplasma faucium* was identified in samples of mucus, umbrella, and arms in both sampling times. This species, lacking a cell wall, is unaffected by many common antibiotics such as penicillin or other beta-lactam antibiotics that target cell wall synthesis. It was first described in 1974 and was regarded as a commensal of the human oral flora and as non-pathogenic until 2009 when it was identified for the first time in some brain abscesses [105]. *Mycoplasma* spp., including *M. faucium* were isolated from wounds of sea lions (*Zalophus californianus*) undergoing rehabilitation in California and related to the death or disease of some animals [106]. In this framework, the identification of *M. faucium* in all jellyfish samples (different compartments and sampling months) indicates a strict relationship with *R. pulmo*, which may therefore represent a vector of pathogenic species. However, the nature of the association between *Mycoplasma* and *Spiroplasma* bacteria and jellyfish calls for further investigation.

The majority of Proteobacteria identified in *R. pulmo* belong to the following three classes: Alphaproteobacteria, Epsilonproteobacteria, and Gammaproteobacteria. In particular, the classes Gammaproteobacteria and Epsilonproteobacteria were the most abundant in the three compartments in T1 samples (July 2016), even though reduced abundance values were accounted for the umbrella compartment. Also in T1, the class of Gammaproteobacteria was most represented by the genera *Coxiella* in mucus samples, *Vibrio* in umbrella samples, and *Endozoicomonas* in arms. This trend was similar in T2 (February 2017) except for umbrella compartment, when the Alphaproteobacteria class were the most abundant taxon, with the genus *Lentibacter* (*L. algarum).* Among the roseobacters, *Lentibacter* spp. are reported to be abundant (up to 30%) in coastal and estuarine waters [107], and after the recent isolation of *L. algarum* from massive green algae bloom in coastal water, it has been repeatedly identified in other geographical areas [108,109].

With regard to the genus *Coxiella*, (Gammaproteobacteria), this includes pathogens like *Coxiella burnetii*, the causative agent of Q-fever, also found in marine mammals, and *Coxiella cheraxi,* a lethal pathogen of the freshwater Australian crayfish *Cherax quadricarinatus* [110,111]. Further analyses are required to know whether the *Coxiella* sp. detected mainly in the mucus compartment of *R. pulmo* belongs to one of those species, or if it represents a new taxon, since the phylogenetic analysis (data not shown) did not help us to clarify this aspect.

Among the *Endozoicomonas*, *Endozoicomonas atrinae* has been identified in samples of arms in both T1 and T2. This bacterial species was isolated for the first time from the intestine of a comb pen shell *Atrina pectinata* in 2014 [112]. Since a great abundance of *Endozoicomonas* spp. was found in healthy invertebrates in absence of disease or manifest injuries, these bacteria have not been considered pathogens for several marine organisms [113,114]. *Endozoicomonas* species have been identified from sponges [115], corals [116,117], and bivalve shells [112]. *Endozoicomonas* genomes suggest symbiotic functional relationships between the bacteria and their host, relative to the potential transfer of carbohydrates, amino acids, and proteins [118]. Recently, the anthozoan corals *Stylophora pistillata*, *Pocillopora verrucosa*, and *Acropora humilis* [118] and the medusozoan jellyfish *Mastigias* cf. *papua* and *Tripedalia* cf. *cystophora* [54] were found in association with *Endozoicomonas*, thus suggesting an old evolutionary origin of the *Endozoicomonas*–cnidarian relationship. However, despite of their widespread occurrence, the functional interactions of these bacteria with their hosts still remain unknown.

In the *R. pulmo* jellyfish umbrella, the genus *Vibrio* prevailed among Gammaproteobacteria. Luminous *Vibrio* spp. have been already isolated from sponges [119] and from colonial invertebrates (hydroids, bryozoans) with chitinous structures [85,120,121]. *Vibrio* species, including *V. xuii* and *V. harveyi,* were also found associated with the digestive cavity of the jellyfish *C. tuberculata* [51]. In the present study, *Vibrio anguillarum* was exclusively associated with jellyfish mucus and umbrella compartments from July samples (T1). This species is infamously known as a major bacterial pathogen affecting more than 50 fresh and salt-water fish species, bivalves and crustaceans, responsible for vibriosis, broadly defined as secondary septicemia following infection [122,123]. Infection through skin as well as ingestion through contaminated water or food may cause vibriosis in wild and reared animals. Because of its high morbidity and mortality rates, this disease is responsible for severe economic losses worldwide. Temperature represents a key limiting factor controlling abundance of several *Vibrio* species [123]. In the laboratory, *V. anguillarum* grows rapidly at temperatures between 25 and 30 °C [122]. The non-recovery of *V. anguillarum* in T2 winter samples might be explained either by a reduction in population size through direct temperature-mediated mortality or by the population entering the so-called viable but non-culturable (VBNC) state, a reversible survival strategy of metabolic quiescence [124,125]. Several *Vibrio* spp. are indeed temperature-sensitive bacteria, requiring warm waters for recovering from VBNC state and for rapid population outbreaks, making vibriosis typical summer diseases [122,126]. In this framework, the current scenario of ocean warming may lead to increasing worsening of vibriosis incidence in wild and farmed aquatic organisms [127] and more generally, to increasing disease risks for marine and terrestrial biota, including humans [128]. The increasing sea surface temperature will lead to increase *Vibrio* spp. abundance in coastal waters [129], with potential severe consequences on human health: as a case in point, *V. anguillarum* resulted already associated with human illness [130]. Overall, the incidence of *Vibrio*-associated illnesses is increasing worldwide and especially in European countries, where recreational activities (swimming/bathing) are common in coastal areas [131,132,133]. Ocean warming may cause interacting mechanisms to drive increased health risks for humans: A temperature-dependent increase of jellyfish outbreaks in coastal waters [1] will eventually concur to promote increased abundance of jellyfish-associated bacteria (including pathogens), ultimately leading to enhancement of physiological stresses of wild and farmed fish populations [25]. Throughout jellyfish outbreaks, jellyfish mucus—a preferential substrate for most bacteria—is released in large amount in the water column, so that a strong, negative impact on human health might be hypothesized. Further studies will be required to determine the extent of risks of bacterial disease for human health, particularly in tourist hot spots characterized by jellyfish proliferations.

The “holobiome concept” hypothesizes a strong interplay of commensal and/or mutualistic relationships between associated microorganisms and their host, supporting fitness, health and homeostasis of the meta-organism ([39]; but see also [134] for an historical review). In the jellyfish–microbiome association, some less represented taxa may play an important role. For instance, the genera *Streptococcus* and *Staphylococcus* (Firmicutes) were found only in arms of T2 samples. Both strains were also found in the jellyfish *Aurelia aurita* [135]. In particular, *Streptococcus* strains are supposed to have a role in the prevention of the growth of potential pathogens, through mechanisms of bacterial cell-cell paracrine communication or “quorum sensing”, to control the production of toxins as known in *Streptococcus* [136,137].

The class Epsilonproteobacteria includes a large group of host-associated organisms as well as free-living bacteria, recovered from hydrothermal vents and cold seep habitats. In the digestive tract of organism could be both symbionts (e.g., *Wolinella* spp.) and pathogens (e.g., *Helicobacter* spp., *Campylobacter* spp.). Their ecological key role was completely underestimated in the past [138] and only in last decades they have been discovered to inhabit deep sea and to be connected with S cycle, to be able to reduce nitrate and nitrite [139] and to mediate the Calvin-Benson cycle to fix CO_2_ [140,141]. Our 16S amplicon sequencing analysis revealed several Epsilonproteobacteria associated with *R. pulmo*, suggesting they could have multiple functions with their jellyfish host. However, further studies are needed to verify the consistency of the association in other areas of the Mediterranean and to clarify the nature of the metabolic relationships with *R. pulmo*.

Last but not least, the high bacterial diversity evidenced in the different compartments of *R. pulmo* opens a new scenario in the exploitation of the microbial metabolic pathways for future sustainable biotechnological processes. Besides novel enzymes for specific purposes, the associated microorganisms can indeed be exploited also for novel or improved biotechnological purpose. In this framework, the discovery of new antibiotics and anticancer compounds and exploring the high repertoire of microorganisms associated with *R. pulmo* represent a challenge toward this direction.

## 4. Materials and Methods

### 4.1. Animals, Collection, and Sample Preparation

A total of 40 specimens of *R. pulmo* jellyfish were collected by scuba diving (depth range = 1–4 m) in summer 2016 (T1: July, 04, 14, 19, and 28, 2016) and winter 2017 (T2: February, 02, 09, 15, and 27, 2017) at Ginosa Marina in the Gulf of Taranto (Ionian Sea 40°25.7′ N, 16°53.1′ E; Italy), where recurrent and high-density blooms of *R. pulmo* occur [61]. Sampling was always carried out in daytime around noon (11.00–13.00 h). At each of the eight sampling dates, five *R. pulmo* specimens were individually collected, separately stored within DNA free sterile containers at 5 °C, and rapidly transported to the laboratory within the following 3 h. Jellyfish were then measured and washed with sterile seawater (0.2 μm pre-filtered and autoclaved; see Kos Kramar et al. [38]). Manipulative stress induced jellyfish to produce release of mucus (M) that was collected with a sterile glass pipette [39]. After mucus collection, the five jellyfish from each sampling were dissected with the oral arms detached from the umbrella and both these compartments were homogenized in a sterile Waring blender. The mucus (M), umbrella (U), and oral arms (A) from each of the five jellyfish specimens were pooled in order to obtain three distinct pools (M, U, A) at each sampling date. Each pooled compartment was then used for the 16S rDNA sequencing of the associated microbial community [22], the estimation of culturable bacteria abundance (as colony forming unit CFU count) and the evaluation of microbial metabolic utilization of carbon sources [39].

### 4.2. Microbiological Analyses

To estimate the culturable heterotrophic bacteria abundance, 100 μL of each sample and appropriate decimal dilutions (10^−1^, 10^−2^, 10^−3^, 10^−4^, 10^−5^) were plated in triplicate onto Marine Agar 2216. The culturable bacteria were evaluated after incubation in the dark at 22 °C for 7 days according to the colony forming units (CFU) method [121,142,143].

### 4.3. BIOLOG EcoPlate Inoculation and Incubation

Four replicates of each pooled compartment (M, A, U) each deriving from five specimens of *R. pulmo* from July 2016 (T1: July, 04, 14, 19, and 28, 2016) and February 2017 (T2: February, 02, 09, 15, and 27, 2017) samplings were screened for detection of the temporal potential metabolic utilization of carbon sources by the jellyfish-associated microorganisms through the BIOLOG ECO plate system (BIOLOG Inc., Hayward, Calif.). This is a standardized method applied to soil and aquatic environmental samples [144,145] in order to uncover the minimal cooperative communities of microorganisms associated to different environmental matrixes and substrates [146]. The tool is based on the degradation capability of 31 of the most useful carbon sources (8 amino acids, 9 carbohydrates, 10 carboxylic and acetic acids, and 4 polymers), by using a redox-sensitive, tetrazolium indicator of microbial respiration [147].

The plates were incubated with 150 µL of sample in each well at 22 °C, according to optimal range (18–24 °C) for mesophilic bacteria [148] for 1 week. Absorbance was measured after 24, 48, 72, 96, 120, 144, and 168 of incubation hours, 120 h of incubation was the optima range of optical density and then it was used for statistical analyses, in accordance with to Gryta et al. [146]. By using a plate reader (Microplate Reader model 3550; Bio-Rad, Richmond, Calif.), the optical density (OD) values were measured at a wavelength of 590 nm. The increase in OD values for the well represents an indicator of the growth of microbial communities able to degrade each specific substrate in the plate [149,150,151].

### 4.4. DNA Extraction

Pooled samples (eight for each of the three jellyfish compartments M, A, U) were gathered over the course of field samplings carried out in July 2016 and February 2017 for 16S amplicon sequencing analyses. All pools were stored at −20 °C, and then lyophilized (FreeZone^®^ 12 L; Labconco, Kansas City, MO). Three hundred milligrams of each freeze-dried sample were subjected to the DNA extraction using the FastDNA SPIN kit for soil (BIO 101, Carlsbad, CA) according to the manufacturer’s instructions. Qualitative and quantitative DNA assessment was carried out using the PicoGreen^®^ dsDNA quantitation assay (Invitrogen, Carlsbad, California) and agarose gel (1%) electrophoresis. DNA extraction blanks (sterile distilled water) were prepared and processed together with the jellyfish samples in order to exclude any contaminations related to the extraction reagents and procedure.

### 4.5. 16S rDNA Library Preparation and Sequencing

The microbial DNA extracted from each sample was used as template for the 16S rDNA library preparation (as described in Basso et al., [39]). The hypervariable regions V5–V6 of the 16S ribosomal RNA (rRNA) gene were chosen as amplification targets. Equimolar quantities of the purified obtained amplicons were pooled and subjected to 2 × 250 bp paired-end sequencing on the Illumina MiSeq platform. Together with the samples, a phage PhiX genomic DNA library was sequenced in order to increase the genetic diversity, as required by the MiSeq platform. All the sequencing raw data have been submitted at NCBI SRA repository (SRA accessions PRJNA492850 (run accession: SRR7894431, SRR7894430, SRR7894423, SRR7894422, SRR7894419, SRR7894418, SRR7894417, SRR7894421, SRR7894424, SRR7894427, SRR7894426, SRR7894429), and PRJNA615778).

### 4.6. Taxonomic and Phylogenetic Analyses

The obtained Illumina MiSeq reads were analyzed by using a bioinformatic workflow relying on the ASVs (Amplicon Sequence Variants) inference and their taxonomic classification. In particular, the Nextera adaptors and PCR (Polymerase Chain Reaction) primers were trimmed by using cutadapt [152] and avoiding any quality trimming in order to not influence the following denoising procedure. The obtained ASVs were taxonomically annotated in BioMaS by using the release 11.5 of the RDP database [153,154] and the NCBI taxonomy, as 16S rRNA reference collection and taxonomy, respectively. In particular, the query sequences were aligned to the reference collection by using bowtie2 [155] and the resulting alignments were filtered according to query coverage (≥70%) and identity percentage (≥90%).

The phylogenetic inference was achieved by using the *align-to-tree-mafft-fasttree* plugin: A multiple sequence alignment of ASVs sequences was obtained by using MAFFT [156] and the phylogenetic tree was inferred by applying the maximum-likelihood procedure implemented in Fasttree 2 [157]. Statistical comparisons between jellyfish compartments and sampling periods (T1 and T2) were performed by using DESeq2 [158]. Alpha (Shannon index (H’) [159] and Faith Phylogenetic index (PD) [160]) and Beta diversity (based on Bray–Curtis Dissimilarity matrix [161]) analysis were performed by using the phyloseq [162] and vegan package R packages [163]. PERMANOVA and SIMPER analysis (both with 999 permutations) were used to test differences between groups and infer the ASVs contribution in dissimilarity between groups, respectively.

### 4.7. Statistical Analyses

The Kruskal–Wallis and the Dunn post-hoc tests were used to compare the alpha diversity indices between jellyfish compartments (M, A, U) in the same sampling period (T1 = July 2016; T2 = February 2017). The comparisons between T1 and T2 in a specific jellyfish compartment were achieved by using the Wilcoxon test. The differences in i) culturable bacteria abundance (as colony forming unit CFU count) and ii) optical densities related to carbon sources utilization of microbial communities associated in T1 and T2 with specific jellyfish compartments were assessed by univariate and multivariate PERMANOVA analyses. Data on abundance of culturable bacteria and potential metabolic activities of microbial communities were based on Euclidean distances, using 9999 random permutations of the appropriate units [164]. The experimental design consisted of two factors: sampling month (T, as random factor with 2 levels) and jellyfish compartment (C, as fixed factor with 3 levels), *n* = 4. When significant differences were encountered (*p* ≤ 0.05), post-hoc pairwise tests for the fixed factor were carried out to ascertain the consistency of the differences among compartments. When the number of unique permutations was restricted in the pairwise tests, we obtained *p* values from Monte Carlo [164]. Canonical analysis of principal coordinates (CAP) was performed in order to plot the optical densities related to carbon sources utilization of microbial communities associated with *R. pulmo* compartments [165]. The analyses were performed using the software PRIMER v6 [166].

## 5. Conclusions

Our results indicate that body surfaces and mucous secretions of *R. pulmo* represent suitable substrates for the settlement and growth of diverse communities of marine microorganisms. Microorganisms associated with jellyfish mucus are characterized by a great diversity, abundance (as colony forming unit CFU count), and metabolic activities related to carbon sources utilization. However, some bacteria may establish mutualistic relationships with the jellyfish (e.g., *Endozoicomonas*) whereas other bacteria could represent a threat to the health of marine organisms and humans (e.g., *Coxiella* and *Vibrio*). Moreover, during jellyfish outbreaks, the microbial community could proportionally increase their abundance and spread in the surrounding aquatic environment. Due to the fast turnover rates and related fast responses to ecosystem change, jellyfish are candidates as early warning indicators of impacts potentially affecting the structure of trophic webs (Marine Strategy Framework Directive 2008/56) and, as a corollary, the microbiological quality of coastal habitats. Further manipulative investigations will be required to gather information on the potential role of jellyfish on the spread of pathogens in coastal systems, overall improving our understanding of marine biodiversity dynamics and of the related ecological processes. Finally, the high microbial diversity observed in the examined compartments of *R. pulmo* requires further investigation on account of the biotechnological relapses related to microbial exploitation for drug discovery and bioprospecting of new natural products, including antibiotics and anticancer compounds.

## Figures and Tables

**Table 1:** Results of PERMANOVA testing for differences in the associated heterotrophic bacteria abundance (as colony forming unit (CFU) counts) and the optical density due to the potential of specific microbial population to utilize different carbon sources among compartments in July (T1: July, 04, 14, 19, and 28, 2016) and February (T2: February, 02, 09, 15, and 27, 2017), *n* = 4.

Source	df	MS	Pseudo-F	P(perm)	MS	Pseudo-F	P(perm)
		Heterotrophic Abundance			Optical Density		
C	2	4.26 × 10^8^	1.86		22.41	1.14	
T	1	4.17 × 10^8^	13.72		10.46	9.18	
CxT	2	2.29 × 10^8^	7.55	***	19.71	17.29	***
Res	12	3.04 × 10^7^			1.14		
Tot	17						

C—Compartment; T—Time; Res—residual; Tot—total; df— degrees of freedom; MS—mean squares; Pseudo-F—F critic; P(MC) —probability level after Monte Carlo simulations; ***—*p* < 0.001.

**Table 2:** Results of the pairwise tests contrasting the *R. pulmo* associated heterotrophic bacteria abundance (as colony forming unit CFU counts) and the bacterial optical density due to the potential of specific microbial population to utilize different carbon sources among umbrella (U), oral arms (A) and mucus (M) in July (T1) and February (T2).

	t	P(MC)	t	P(MC)	t	P(MC)	t	P(MC)
		Heterotrophic Abundance			Optical Density			
	T1		T2		T1		T2	
M vs U	3.61	*	5.97	**	4.56	***	3.72	*
M vs A	2.1	*	2.02	ns	5.67	***	3.61	*
U vs A	3.51	**	2.91	*	3.60	**	3.84	*

P(MC)—probability level after Monte Carlo simulations; t—pairwise tests. *—*p* < 0.05; **—*p* < 0.01; ***—*p* < 0.001; ns—not significant.

**Table 3:** Metabolic utilization of 31 different carbon sources by the microbial community associated with oral arms (A), mucus (M), and umbrella (U), of *R. pulmo* in July (T1) and February (T2).

	Oral Arms		Mucus		Umbrella	
	T1	T2	T1	T2	T1	T2
γ−Hydroxybutyric Acid	+	+	−	+	−	−
d−Glucosaminic Acid	+	+	−	+	−	−
d−Galacturonic Acid	+	+	+	+	−	−
l−Phenylalanine	+	+	+	−	−	−
d−Xylose	+	−	−	+	−	−
Pyruvic Acid Methyl Ester	+	−	−	−	−	−
l−Threonine	−	+	+	−	−	−
Glucose−1−Phosphate	−	+	+	+	−	+
Glycyl−l−Glutamic Acid	−	+	+	+	−	−
d.l−α−Glycerol Phosphate	−	+	−	−	−	−
l−Arginine	−	+	+	+	−	−
Tween 40	−	+	+	+	+	−
N−Acetyl−d−Glucosamine	−	+	−	−	−	−
d−Mannitol	−	+	−	−	−	−
Glycogen	−	+	+	−	−	−
β−Methyl−d−Glucoside	−	−	+	+	−	−
d−Galactonic Acid γ−Lactone	−	−	−	−	−	−
l−Asparagine	−	−	+	−	−	−
i−Erythritol	−	−	+	−	−	−
2−Hydroxy Benzoic Acid	−	−	−	+	−	−
Tween 80	−	−	−	−	+	−
4−Hydroxy Benzoic Acid	−	−	−	−	−	−
l−Serine	−	−	+	+	−	−
α−Cyclodextrin	−	−	+	+	−	−
Itaconic Acid	−	−	−	+	−	−
d−Cellobiose	−	−	+	+	−	−
α−Ketobutyric Acid	−	−	+	−	−	−
Phenylethyl−amine	−	−	−	−	−	−
α−d−Lactose	−	−	+	+	−	−
d−Malic Acid	−	−	−	−	−	−
Putrescine	−	−	−	+	+	−
